# Understanding the Situated Roles of Electronic Medical Record Systems to Enable Redesign: Mixed Methods Study

**DOI:** 10.2196/13812

**Published:** 2019-07-09

**Authors:** Samar Helou, Victoria Abou-Khalil, Goshiro Yamamoto, Eiji Kondoh, Hiroshi Tamura, Shusuke Hiragi, Osamu Sugiyama, Kazuya Okamoto, Masayuki Nambu, Tomohiro Kuroda

**Affiliations:** 1 Department of Social Informatics Graduate School of Informatics Kyoto University Kyoto Japan; 2 Division of Medical Information Technology and Administration Planning Kyoto University Hospital Kyoto Japan; 3 Department of Gynecology and Obstetrics Graduate School of Medicine Kyoto University Kyoto Japan; 4 Center for Innovative Research and Education in Data Science Kyoto University Kyoto Japan; 5 Preemptive Medicine and Lifestyle Related Diseases Research Center Kyoto University Hospital Kyoto Japan

**Keywords:** computerized medical record systems, physicians' offices, design, role, prenatal care, Japan, observational study

## Abstract

**Background:**

Redesigning electronic medical record (EMR) systems is needed to improve their usability and usefulness. Similar to other artifacts, EMR systems can evolve with time and exhibit situated roles. Situated roles refer to the ways in which a system is appropriated by its users, that is, the unintended ways the users engage with, relate to, and perceive the system in its context of use. These situated roles are usually unknown to the designers as they emerge and evolve as a response by the users to a contextual need or constraint. Understanding the system’s situated roles can expose the unarticulated needs of the users and enable redesign opportunities.

**Objective:**

This study aimed to find EMR redesign opportunities by understanding the situated roles of EMR systems in prenatal care settings.

**Methods:**

We conducted a field-based observational study at a Japanese prenatal care clinic. We observed 3 obstetricians and 6 midwives providing prenatal care to 37 pregnant women. We looked at how the EMR system is used during the checkups. We analyzed the observational data following a thematic analysis approach and identified the situated roles of the EMR system. Finally, we administered a survey to 5 obstetricians and 10 midwives to validate our results and understand the attitudes of the prenatal care staff regarding the situated roles of the EMR system.

**Results:**

We identified 10 distinct situated roles that EMR systems play in prenatal care settings. Among them, 4 roles were regarded as favorable as most users wanted to experience them more frequently, and 4 roles were regarded as unfavorable as most users wanted to experience them less frequently; 2 ambivalent roles highlighted the providers’ reluctance to document sensitive psychosocial information in the EMR and their use of the EMR system as an accomplice to pause communication during the checkups. To improve the usability and usefulness of EMR systems, designers can amplify the favorable roles and minimize the unfavorable roles. Our results also showed that obstetricians and midwives may have different experiences, wants, and priorities regarding the use of the EMR system.

**Conclusions:**

Currently, EMR systems are mainly viewed as tools that support the clinical workflow. Redesigning EMR systems is needed to amplify their roles as communication support tools. Our results provided multiple EMR redesign opportunities to improve the usability and usefulness of EMR systems in prenatal care. Designers can use the results to guide their EMR redesign activities and align them with the users’ wants and priorities. The biggest challenge is to redesign EMR systems in a way that amplifies their favorable roles for all the stakeholders concurrently.

## Introduction

### Enabling Redesign by Understanding the Situated Roles of an Electronic Medical Record System

The usability and usefulness of electronic medical record (EMR) systems are critical for their acceptance and effective use [1-3]. Accordingly, multiple user and usability studies were conducted with the aim of refining the systems’ functional and nonfunctional specifications [4-15]. Previous studies used interviews, surveys, focus groups, and observations to identify the needs of EMR users and the issues they encounter when using EMR systems. However, EMR users may have needs that they are not aware of or cannot articulate. Moreover, the experts’ verbal description of their work could be inconsistent with how they perform it in the field [16].

**Figure 1:**
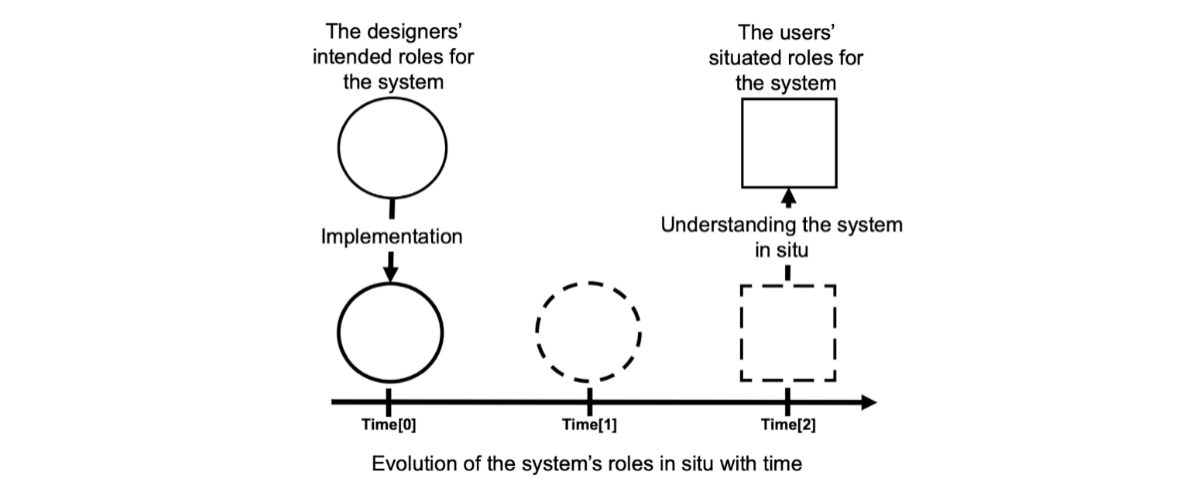
Approach for identifying system redesign opportunities.

To address these limitations, we adopted a novel approach for finding EMR redesign opportunities. The approach is based on the idea of *redesigning from appropriation* [17]. Following this approach, we analyzed the EMR system as an artifact that evolves with time and exhibits situated roles. Situated roles refer to the ways in which a system is appropriated by its users, that is, the unintended ways the users engage with, relate to, and perceive the system in its context of use. The conceptual approach is depicted in Figure 1. Multiple EMR situated roles can exist; for example, one could use it as an explanation support tool to communicate information to a patient during a consultation or as an excuse to take a break from the conversation. These situated roles are usually unknown to the designers as they emerge with time as a response by the users to a contextual need or constraint. Understanding the situated roles of the system and the users’ attitudes regarding them could enable user-centered redesign opportunities.

We applied our approach in prenatal care settings, a unique health care setting that does not fit into the common clinician-patient scheme where EMR systems are usually studied. Prenatal care is the periodic care that a pregnant woman receives during her pregnancy. Unlike other health care settings where the aim is to address a patient’s health problems, the main goal of prenatal care is the prevention and early detection of diseases that can affect the pregnant woman and her fetus(es) [18]. Therefore, prenatal care is the setting in which we collect information about the health of an individual for the first time. The effective use of EMR systems in antennal care is needed if we aim to have complete longitudinal health records.

### Purpose of This Study

The purpose of this study was to identify EMR redesign opportunities in prenatal care settings. We achieved this purpose by answering the following research questions:

- What are the situated roles of EMR systems in prenatal care settings?
- Do users want to experience the situated roles more, or less, frequently?
- How important are the different situated roles to the users?

Using our results, designers can align their EMR redesign activities with the wants and priorities of EMR users in prenatal care.

### Prenatal Care in Japan

In Japan, prenatal care is standardized by the Japan Society of Obstetrics and Gynecology and the Japan Association of Obstetricians and Gynecologists. Most of the pregnant women in Japan attend the recommended regular prenatal checkups. Women with uncomplicated pregnancies usually receive 14 checkups. The checkups start around their 8th week of pregnancy and finish 1 week after childbirth [19,20].

During prenatal care, almost all pregnant women carry the *Boshi Kenko Techo*, a paper-based maternal and child health (MCH) handbook. The MCH handbook is filled and reviewed by the prenatal care providers. The MCH handbook contains information about the woman’s pregnancy and the child’s development and health [20,21].

Japanese women can receive midwife-led (MW-led) prenatal care or obstetrician-led (OB-led) prenatal care. Previous studies have found that pregnant women in the MW-led care group gave higher ratings to their care satisfaction and their perception of woman-centered care [22]. These results highlight the different responsibilities of the obstetricians and the midwives. The obstetricians’ focus is mostly biomedical, whereas the midwives’ focus is to promote self-care.

In Japan, the adoption of EMR systems has been steadily increasing since 2005 [23]. By 2020, the EMR adoption rate is expected to reach 90% for general hospitals [24]. Although EMR systems are regularly used in Japanese prenatal care, little is known about their use and about the attitudes of the prenatal care providers regarding them.

### Redesigning from Appropriation

In a proposed model for artifact study, Fleming talked about the function of an artifact being one of its 5 basic properties [25]. He proposed that the function “embraces both the uses (intended functions) and the roles (unintended functions) of the object in its culture.” He also noted that functional analysis would have to involve the discussion of the human and their artifact-associated behavior.

In a similar vein, multiple studies in computer-supported cooperative work shed light on these unintended functions through the concept of appropriation. Once deployed in their contexts of use, artifacts or technologies are appropriated by their users [26,27]. Appropriation is “the way technologies are adopted, adapted and incorporated into working practice” [28]. Dourish presented the concept of appropriation as a broader view of customization, one that includes users “making use of the technologies for purposes beyond those for which it was originally designed, or to serve new ends” [28]. In this sense, Dourish noted that appropriation lies at the intersection of workplace studies and design and that understanding how technologies are appropriated is critical to developing them [28].

It has been suggested that understanding the ways in which a technology is appropriated is important to improve its design process [29,30]. Carroll argues that the appropriation of technologies is part of their design process. As the users appropriate a technology, they play a crucial role in completing its design [17]. Carroll also proposed improving the technologies’ design by harvesting the users’ needs from their appropriation activities. By deriving requirements from the appropriated technology, the designer would “design from appropriation” and involve the users as co-designers in an evolutionary design approach [17]. On a similar note, Fischer described the “impossibility of complete coverage” as one of the biggest design challenges for designing high-functionality environments. To address this challenge, he proposed “viewing the systems as open-ended and continuously adapted by the people who use them in their day-to-day work” [31].

In their work on persuasive technologies, Krischkowsky et al argued that the study of the unintended uses of a technology is critical to counteract undesired consequences [32]. Furthermore, we can find various works in the fields of human-computer interaction [31] and design [33] that aim to understand the appropriation of technologies in their contexts of use.

### Roles of Electronic Medical Record Systems

Chase et al [34] examined the roles of electronic health record (EHR) systems regarding the collaboration between care providers. They identified 4 general EHR roles:

- Repository: the EHR allows the providers to have all the needed data in one place.
- Messenger: the EHR enables information transfer between the providers.
- Orchestrator: the EHR ensures that the right person is doing the right thing at the right time.
- Monitor: the EHR allows the identification of gaps in treatment and provides a benchmark for performance evaluation.

Through these roles, they described the ways in which the EHR system supports or hinders collaboration. Unlike this study, the roles were not extracted with the aim of *redesigning from appropriation* and, thus, were not translated into design recommendations.

## Methods

### Overview

**Figure 2:**
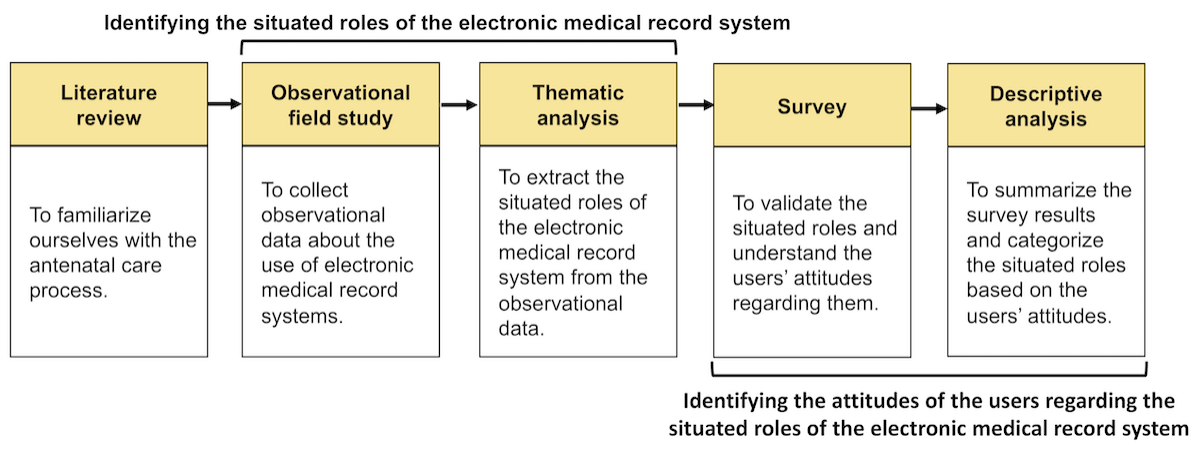
The applied methods.

In this study, we used mixed research methods in an exploratory sequential manner—a qualitative study followed by a quantitative study. We used the results from the qualitative method to inform the quantitative method. The sequence of the applied methods is shown in Figure 2.

After familiarizing ourselves with the prenatal care process, we conducted a field-based observational study at a Japanese prenatal care clinic. We analyzed the data following a thematic analysis approach to identify the different situated roles that the EMR system plays. Then, we administered a survey to the prenatal care staff to understand their experiences and attitudes regarding the situated roles of the EMR system. Finally, we analyzed the survey data and categorized the situated roles of the EMR system based on the users’ experiences and attitudes. In the following sections, we describe in detail how we conducted each step of the study.

### Literature Review—Familiarization With the Prenatal Care Process

To rapidly gather a large corpus of knowledge and gain an initial understanding of the prenatal care process, we conducted a review targeting the existing literature on the prenatal care process and guidelines for obstetrical practices in Japan [18,19,20,21,22]. We validated our initial understanding of the process by discussing it with a practicing obstetrician at the prenatal care clinic.

### Identifying the Situated Roles of the Electronic Medical Record System

#### Data Collection: Observational Field Study

One researcher observed a team of obstetricians and midwives providing prenatal care services at an outpatient clinic in a Japanese university hospital. In the observed clinic, a total of 5 obstetricians and 10 midwives provide prenatal care services. After obtaining the approval of 3 obstetricians to observe checkups during their shifts, the researcher conducted the observations by visiting the prenatal care outpatient clinic twice a week over a period of 3 weeks.

At the beginning of the checkups, the obstetricians explained to the pregnant women and their companions the reasons for the researcher’s presence in the clinic. The obstetricians also asked the pregnant women and their companions if they accept having the researcher observe and take notes during the checkup.

After the pregnant women and their companions granted their approval, the researcher directly observed the prenatal care checkups and took notes using pen and paper. The notes contained descriptions of the interactions that took place around the EMR system, key information relating to the women’s course of pregnancy (pregnancy week, pregnancy type, and pregnancy number), sketches of the room layout and the EMR screen, and quotes and impressions from the conversations that took place around the EMR system.

**Figure 3:**
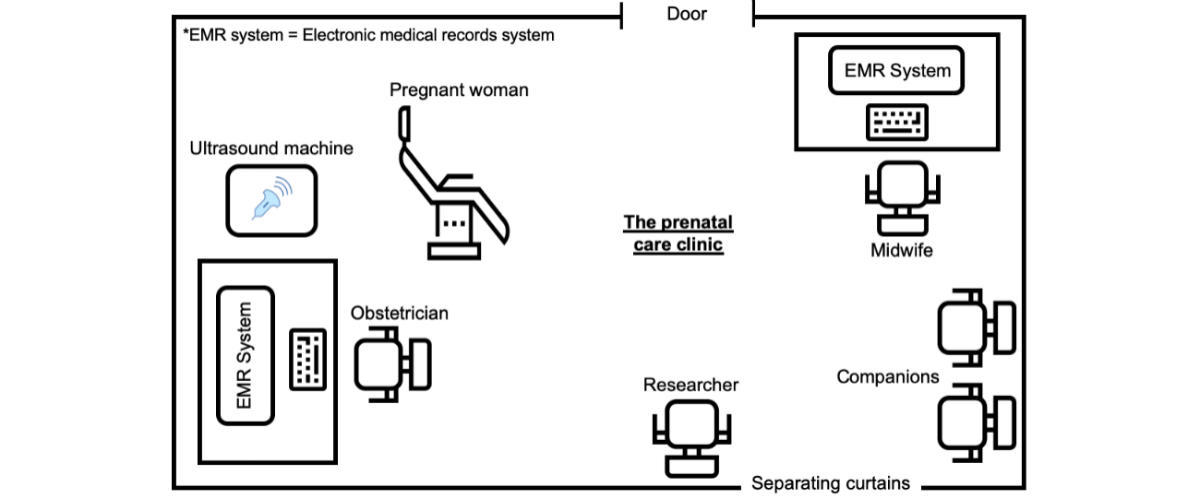
The observed prenatal care clinic.

In the observed clinic, there were 2 desks with computer terminals, as shown in Figure 3. One desk was used by the obstetrician and the other by the midwife. The room layout was *semi-inclusive patient controlled*. The pregnant women could see the EMR screen by moving the direction of their gaze [35].

During the observations, the researcher did not engage in conversations with the present parties. After the pregnant women and their companions left the clinic, the researcher asked the clinical staff questions to clarify certain occurrences. The researcher asked for information about the software that the staff used in addition to the EMR software. The researcher also asked for explanations as to why certain things were done in certain ways, for example, (1) using an image snipping tool, (2) bolding and changing the color of certain text, or (3) copying information from EMR notes and pasting them in other notes. In addition, the researcher inquired about artifacts that were used during the checkups, such as reference books that the staff used and paper files that the pregnant women exchanged with the staff.

In total, the researcher observed a team of 3 obstetricians and 6 midwives performing 37 prenatal care checkups for 35 different pregnant women between the eighth and 33rd week of their pregnancy.

#### Data Analysis: Thematic Analysis

After each observation, the field notes were transcribed and imported into QDA Miner, a qualitative data analysis tool made by Provalis Research [36]. After the observations were completed, the data were analyzed by 3 researchers following the 6 phases of thematic analysis described by Braun et al [37]:

- Familiarization with the data: In the beginning of the analysis process, we read and discussed the data multiple times to familiarize ourselves with it.
- Coding the data: The coding process was conducted over 3 iterations in which the codes were extended and refined. The process is described below.

While familiarizing ourselves with the data, we found that the interactions with the EMR system fall into 4 main categories: (1) interactions that support the communication, (2) interactions that hinder the communication, (3) interactions that support the clinical process, and (4) interactions that hinder the clinical process. These categories were mapped into 4 initial codes and were used in the first coding iteration.

After the first coding iteration, we noted more specifically how the EMR system supports/hinders the communication/process. These more detailed descriptions were used to code the data in the second coding iteration.

After the second coding iteration, we extended the codes to reflect aspects that could not be captured in the original codes and we merged codes together when their contents overlapped. Using these extended and refined codes, we conducted our third coding iteration.

- Searching for the themes: after the coding was completed, 2 researchers examined the codes to see which ones could fit together under one theme. A theme was considered to be any set of codes that captures a significant or interesting unintended way in which the parties interact with the EMR system.
- Reviewing the themes: in this step, we discussed which themes qualify as situated roles of the EMR system. A situated role of the EMR system would be any theme that reflects an unintended way that the users engage with, relate to, and perceive the system in its context of use. When deciding which themes to keep and which themes to discard, we answered the following questions:
  1. Does the theme really reflect an unintended way that the users engage with, relate to, and perceive the system?
  2. Does the theme make sense?
  3. Does the data that we collected support our conclusion?
- Defining and naming the themes: after reviewing the themes, we finally named and clearly defined them to reflect situated roles of the EMR system.
- Producing the report: the situated roles of the EMR system are presented in the Results section.

### Identifying the Attitudes of the Prenatal Care Providers Regarding the Situated Roles of the Electronic Medical Record System

#### Survey Design

After the situated roles were defined and named through the thematic analysis process, we administered a survey for all the prenatal care staff working at the observed clinic. In total, 15 surveys were sent out to 5 obstetricians and 10 midwives. The survey participants included the obstetricians and midwives that we observed in the field study.

First, the purpose of the survey was to validate the situated roles through the experiences of the users. Second, we wanted to understand how frequently the users want to experience the EMR roles and how important is each role to them. Therefore, for each situated role, we asked 3 questions:

1. Currently, how frequently do you experience [role’s definition]?
2. Optimally, how frequently would you experience [role’s definition]?
3. It is important to me that the EMR system does [role’s definition] OR It is important to me that the EMR system does not [role’s definition]

The purpose of the first question was to validate the situated roles through the users’ current experiences. Answers to this type of question were reported using a 6-point Likert scale ranging from very frequently (1) to not at all (6).

The purpose of the second question was to understand how frequently the users wanted to experience each role. Answers to this type of question were reported using a 6-point Likert scale ranging from very frequently (1) to not at all (6).

The purpose of the third question was to understand the importance of each situated role. These statements were formulated based on the roles’ nature. For favorable roles, we asked about the importance of their presence. For unfavorable roles, we asked about the importance of their absence. Answers to these statements were reported using a 4-point Likert scale ranging from strongly agree (1) to disagree (4).

In addition to the questions regarding the situated roles, the respondents were asked for their job title and the number of years they had used the target EMR system.

The survey was designed over several iterations. It was pretested by researchers in medical informatics and human-computer interaction who evaluated the structure, understandability, scales, and formulation of the questions. Finally, the survey was pilot tested with 2 graduate students and refined based on their experience and feedback. The final survey was administered in Japanese.

#### Survey Participants

We received a total of 15 survey responses, 5 from obstetricians and 10 from midwives. Table 1 shows the experience of the participants with the EMR system.

#### Survey Analysis

The purpose of the survey was threefold: (1) validating the situated roles through the experiences of the users, (2) understanding how often the users want to experience each situated role, and (3) understanding how important each situated role is to the users.

##### Validation of the Situated Roles

On the basis of the survey responses, we considered that a situated role is validated if at least one respondent reports experiencing it occasionally.

We also categorized the situated roles into 3 categories reflecting the extent to which they are currently experienced by the users:

1. Frequently: more than half of the respondents experienced the role at least frequently.
2. Occasionally: more than half of the respondents experienced the role at least occasionally.
3. Rarely: more than half of the respondents experienced the role rarely at most.

##### Desired Frequency of the Situated Roles

We categorized the situated roles into 3 categories reflecting the extent to which they are wanted to be experienced by the users:

1. Frequently: more than half of the respondents wanted the role to be experienced at least frequently.
2. Occasionally: more than half of the respondents wanted the role to be experienced at least occasionally.
3. Rarely: more than half of the respondents wanted to experience the role rarely at most.

##### Importance of the Situated Roles

On the basis of the survey responses, we categorized the situated roles into 4 categories reflecting their degree of importance for the users:

1. Very important: at least half of the respondents strongly agree that the presence or absence of the role is important.
2. Important: at least half of the respondents agree that the presence or absence of the role is important.
3. Somewhat important: at least half of the respondents somewhat agree that the presence or absence of the role is important.
4. Not important at all: at least half of the respondents disagree that the presence or absence of the role is important.

**Table 1:** The survey participants.

Participant	Job	Experience with the EMR^a^ system (years)
O1	Obstetrician	1
O2	Obstetrician	1
O3	Obstetrician	7
O4	Obstetrician	4
O5	Obstetrician	7
M1	Midwife	12
M2	Midwife	3
M3	Midwife	12
M4	Midwife	3
M5	Midwife	5
M6	Midwife	13
M7	Midwife	1
M8	Midwife	5
M9	Midwife	3
M10	Midwife	6

^a^EMR: electronic medical record.

## Results

### Situated Roles of the Electronic Medical Record System

In total, we were able to extract 10 distinct situated roles that the EMR system plays in prenatal care settings.

We found 4 situated roles relating to the communication between the providers, the pregnant women, and their companions, namely: (1) the wingman, (2) the accomplice, (3) the third wheel, and (4) the bouncer.

Regarding the clinical process, we found that the EMR system plays 6 different situated roles, namely: (1) the messenger, (2) the summarizer, (3) the assistant, (4) the gossip, (5) the alien, and (6) the bureaucrat. Table 2 shows the situated roles and their definitions.

#### The Wingman

As a wingman, the EMR system supports the care providers in the explanation process.

During the checkups, the clinical staff verbally communicated clinical information to the pregnant women and their companions. This communication helps the pregnant women and their companions understand the current state of the pregnancy and the logic behind clinical decisions. In the observations, the obstetricians used the EMR system as a support tool to provide clinical information and explanations. We observed the obstetricians pointing toward the screen while reading their EMR notes and explaining them. The obstetricians also used automatically generated charts and ultrasound images from their EMR notes to visually communicate information to the pregnant women and their companions.

However, the obstetricians did not always automatically employ this strategy. In one case, while the obstetrician was explaining, the pregnant woman started leaning toward the EMR system’s screen to see the image that the obstetrician was looking at. Only after realizing that the pregnant woman was interested in seeing the image did the obstetrician rotate the monitor in her direction.

#### The Accomplice

As an accomplice, the EMR system helps pause communication with the pregnant women.

We found that the obstetricians used the EMR system as a tool to pause communication with the pregnant women, a strategy which proved particularly useful when their workload was high or in highly emotional situations.

One of the obstetricians expressed the need for a *moment to think* in which they do not have to maintain a conversation with the pregnant women. In such cases, the EMR system served as a tool to pause the conversation and provide them with the needed moment to think.

Moreover, talking about pregnancies, especially complicated ones, could result in highly emotional situations. In this case, the EMR system provided the obstetricians with a *bubble* allowing them to distance themselves from the interaction. In one observed case, the obstetrician had to tell the pregnant woman that her pregnancy must be terminated. This woman had already experienced a pregnancy termination. After receiving the information, the pregnant woman started crying. At that moment, the obstetrician resorted to the EMR system to avoid looking at the pregnant woman and allow her to privately wipe her tears and stop herself from crying. When the obstetrician turned to the EMR system, the midwife left her desk and went toward the pregnant woman with a tissue box in hand. The midwife continued standing next to the pregnant woman while the obstetrician was working on the EMR system.

#### The Third Wheel

As a third wheel, the EMR system distracts the care providers from communicating with the pregnant women.

We found that the obstetricians spent a major part of the checkup time keyboarding and facing the EMR screen. During the obstetricians’ data input time, the pregnant women waited silently in their chair, looked closely at the EMR screen to see what their obstetrician was typing, or tried to initiate a conversation with the obstetrician or their companions.

**Table 2:** The situated roles of electronic medical record systems in prenatal care.

Situated role	Definition
The wingman	Supports the care providers in the explanation process.
The accomplice	Helps pause communication with the pregnant women.
The third wheel	Distracts the care providers from communicating with the pregnant women.
The bouncer	Excludes the pregnant women and their companions from the electronic medical record.
The messenger	Enables the communication of information between the care providers.
The summarizer	Provides a quick summary of the pregnancy’s current state and care course.
The assistant	Facilitates the management and preparation of the checkups.
The gossip	Is not completely trusted with sensitive information.
The alien	Has low learnability, requires recall, and does not support routine tasks.
The bureaucrat	Requires the care providers to halt the clinical process to input data.

While inputting data, the obstetricians responded to the pregnant women in various ways. Most of the time, they responded by turning their heads slightly away from the screen toward the pregnant woman. When the pregnant woman continued to ask questions or tried to engage in conversation, the obstetricians either started to alternate quickly between the screen and her or stopped inputting data and turned their chair away from the desk to face and respond to her. In some cases, they fully rotated their chair, but in most cases, they turned it halfway between their desk and the pregnant woman.

#### The Bouncer

As a bouncer, the EMR system creates an exclusive environment by physically excluding the pregnant women and their companions.

On multiple occasions, we found that the pregnant women and their companions showed interest in looking at the EMR. However, the pregnant women had to actively get closer to the screen while their companions’ assigned chairs were placed too far from the screen, leading most of them to stop trying to look at the screen after a while.

On one occasion, the companion of the pregnant woman stood up to get a better view of the EMR screen. After standing up and realizing that he still cannot get a clear view, he tilted his head and body forward in the direction of the screen. When he realized that, even in this position, he cannot clearly see the contents of the EMR, he went back to his seat. After some time, he got up again, tilted forward toward the screen and went back to his seat, clearly feeling disappointed. A while later, he repeated the same sequence: he stood up, tilted forward, and sat down again. After sitting down, he gazed at the floor, bored and frustrated. Finally, he stood up, moved closer to the pregnant woman and to the EMR screen and remained standing there until the end of the checkup.

#### The Messenger

As a messenger, the EMR system enables the communication of information between the care providers.

In the case of the observed clinic, every pregnancy was cared for by multiple obstetricians and midwives. The rotation of the clinical staff required them to communicate the pregnant women’s health data. The EMR system was the main tool for communicating clinical information to ensure continuity of care. The EMR system, in this case, provided seamless communication between the clinical staff over time and staff rotations.

Conversely, when pregnant women were transferred from other clinics, the team only had access to the paper records that they had brought with them. In this case, the team created new EMRs for the women. However, the previous notes existing in the paper records were not transferred to the newly created EMRs.

Even though the pregnant women and their family members are involved in communicating health-related information to the care providers, they did not have the ability to directly add information into the EMR. During the checkups, through conversations with the pregnant women and notes from the women’s MCH handbooks, the providers gathered information and added them into EMR memos. However, what went into the EMR remained under the full control of the care providers.

In one examination, a pregnant woman, with a history of high blood pressure, brought along a paper containing a list of blood pressure measurements that she self-monitored and recorded. The obstetrician reviewed the measurements and handed the paper back to the woman. Then, the obstetrician wrote a note in an EMR memo regarding the measurements. However, the full list of blood pressure measurements remained out of the woman’s EMR.

#### The Summarizer

As a summarizer, the EMR system provides the care providers with a summary of the pregnancy’s current state and care course.

The EMR system allows the prenatal care providers to have all the health information in one place. On multiple occasions, before calling a pregnant woman into the clinic, the obstetricians quickly navigated through the previous EMR notes to form a mental summary of her current course of pregnancy.

However, the EMR system did not allow for a quick understanding of the current state of the pregnancy. One obstetrician noted, “we would like to see the course of care in one glance. With paper records, it was easier to do that. However, with this system, it takes a lot of clicking and scrolling to get the full image.”

The staff needed the EMR system to act as a summarizer. To achieve that, the obstetricians employed a workaround. To give themselves and the other providers a quick understanding of the care course, the obstetricians emphasized certain parts of their EMR notes by changing the size, boldness, and color of the text.

#### The Assistant

As an assistant, the EMR system facilitates the management and preparation of the checkups.

At the beginning of their shift, using the EMR system, the obstetrician and the midwife viewed the list of scheduled checkups. Knowing the number of checkups, they could estimate the workload for the day. Based on that information, they adapted the speed of their work and the duration of checkups. Moreover, using the scheduled checkups list, the obstetrician and midwife knew who they were examining next and had access to her records before the checkup. Before calling the woman in, they reviewed the previous notes and discussed the current state of the woman’s pregnancy. Using this information, they could form a picture of what care actions they needed to perform once the pregnant woman was called in. By allowing for previous preparation, the EMR system makes the checkups run more smoothly. It eliminates the need for the staff to orient themselves and for the pregnant woman to explain the reason for her visit at the beginning of her visit.

#### The Gossip

As a gossip, the EMR system is not completely trusted with sensitive information. In our analysis, we found that the clinical staff hesitate to include highly sensitive information in the pregnant women’s records because of privacy and legal concerns. In one of our discussions with the staff, one midwife stated,

If we have concerns over some psychosocial issues such as domestic abuse, we note it indirectly in the record. We do not write it literally; we use codes to pass the message to the other clinical staff.

Employing this sort of strategy to document sensitive information implies that the EMR system is not completely trusted by the staff with information that is usually considered private or could be used for legal purposes.

#### The Alien

As an alien, the EMR system (1) has low learnability, (2) requires a high level of recall, and (3) has an interface that is not optimized for routine tasks.

The difficulty of learning how to use the EMR system was one of the problems noted by the obstetricians. One obstetrician mentioned that it took them at least 1 month to get used to the system. Moreover, during a checkup, a staff member walked into the clinic and asked the obstetrician a question regarding the use of the EMR system, which the obstetrician answered by guiding them through the interface.

Furthermore, the EMR system appeared to require a high level of memory recall. The obstetricians frequently paused and tried to recall in which tab a specific field, or note, was placed. The inconvenience of the manual data input was further amplified by an interface design that was not optimized for routine data input and data retrieval tasks.

In addition, the EMR system suffered from performance-related issues. In certain cases, the system would temporarily stop responding or have a slow response time. These issues occurred particularly when the providers opened a new EMR. Even though 90% of the common database queries are usually cached, and the list of patients is previously compiled, opening a new EMR required more than 10 seconds in certain observed cases. This poor performance resulted in obvious frustration and time loss. To counter this issue, some midwives employed a sort of manual caching where they anticipated the need to open the records, opened the records, and let them load before they actually needed them.

#### The Bureaucrat

As a bureaucrat, the EMR system requires the care providers to halt the care process to input data.

During the checkups, the providers continuously collect data from multiple sources and add it into the EMR. Those sources include conversations with the pregnant women and their family members; ultrasound imaging devices; and measuring devices such as blood pressure meters, weighing scales, and measuring tapes. The lack of integration between the medical devices and the EMR system required the providers to manually input most of the data that they collect. To do so, they had to intermittently pause their clinical flow. Below are some examples of occurrences encountered during the observations.

Before entering the clinic, the pregnant women use a blood pressure meter and a weighing scale located in the waiting room. The machines print the measurements on small paper receipts. Once they enter the clinic, the women hand the paper receipts and their MCH handbooks to the midwives. During the checkup, the midwives copy the measurements into the MCH handbook and then input them into the EMR. The process of copying the data could take up to 3 min. After they copy the measurements, the midwives throw the small paper receipts in a trash bin under their desks. On 2 different occasions, during ongoing checkups, the midwives had to look in the trash bin for receipts that they had previously thrown away. In one of those occasions, a nurse had to come in, put gloves on, and help the midwife look inside the trash bin.

In addition, the midwives routinely measure the belly circumference before the obstetricians start to conduct the ultrasound. Using a measurement tape, they measure the belly twice, vertically and then horizontally. After the second measurement, the midwives sometimes retake the first measurement, as that they might have forgotten the first measure. Once they finish measuring, some midwives prepare the women for the ultrasound, turn off the lights and then head back to their desks to input the measures. As this increases the risk of forgetting the measures, other midwives prefer to head fast to their desks, input the measures in the MCH handbook and the EMR, and then return to the woman to prepare her for the ultrasound.

As for the obstetricians, they routinely use 2 ultrasound devices to collect data. After they finish conducting the ultrasounds, they reflect on the results and summarize them inside free-text EMR notes. Then, they add the ultrasound images to the notes. To do so, they manually copy the information from the output of the ultrasound devices into the EMR. In addition, they use an image snipping tool to take screenshots of the ultrasound images and then they paste the images inside the EMR notes.

It is important to note that similarly to the *third wheel*, this role is manifested when the providers input data into the EMR system. However, as a *third wheel*, the EMR system hinders the communication between the providers and the pregnant women, whereas as a *bureaucrat*, the EMR system hinders the clinical workflow.

### Attitudes of the Prenatal Care Providers Regarding the Situated Roles of the Electronic Medical Record System

All the prenatal care staff responded to the survey. In total, we received 15 responses, 10 responses from midwives and 5 responses from obstetricians.

#### Validation of the Situated Roles

To validate the situated roles, we analyzed the responses regarding the users’ current experience. We looked at the midwives’ and obstetricians’ answers separately. All the situated roles were validated as at least one respondent reported experiencing them occasionally. Tables 3 and 4 show the responses of the obstetricians and midwives, respectively. The numbers in the table indicate the number of respondents that chose the option.

To better understand the extent to which different roles are experienced, we assigned them to 3 different categories:

1. Frequently: more than half of the respondents experienced the role at least frequently.
2. Occasionally: more than half of the respondents experienced the role at least occasionally.
3. Rarely: more than half of the respondents experienced the role rarely at most.

The extent to which the respondents experience the situated roles is shown in Table 5.

**Table 3:** The current frequency of experiencing the situated roles as reported by the obstetricians. Participant identifier listed in parentheses.

Situated role	Very frequently (n)	Frequently (n)	Occasionally (n)	Rarely (n)	Very rarely (n)	Not at all (n)
Wingman	0	2 (O3, O5)	2 (O1, O4)	1 (O2)	0	0
Accomplice	0	2 (O1, O5)	0	0	2 (O3, O4)	1 (O2)
Third wheel	0	0	3 (O1, O2, O4)	0	0	2 (O3, O5)
Bouncer	0	0	1 (O4)	0	2 (O1, O3)	2 (O2, O5)
Messenger	1 (O3)	3 (O2, O4, O5)	1 (O1)	0	0	0
Summarizer	2 (O2, O3)	3 (O1, O4, O5)	0	0	0	0
Assistant	0	3 (O1, O2, O3)	1 (O5)	0	0	1 (O4)
Gossip	0	0	3 (O1, O4, O5)	1 (O2)	1 (O3)	0
Alien	0	0	4 (O1, O3, O4, O5)	1 (O2)	0	0
Bureaucrat	0	0	2 (O1, O5)	2 (O2, O3)	0	1 (O4)

**Table 4:** The current frequency of experiencing the situated roles as reported by the midwives. Participant identifier listed in parentheses.

Situated role	Very frequently (n)	Frequently (n)	Occasionally (n)	Rarely (n)	Very rarely (n)	Not at all (n)
Wingman	0	1 (M8)	3 (M1, M6, M7)	2 (M4, M5)	1 (M2)	3 (M3, M9, M10)
Accomplice	0	0	7 (M1, M3, M4, M5, M6, M7, M8)	1 (M2)	2 (M9, M10)	0
Third wheel	1 (M3)	0	3 (M1, M4, M6)	2 (M2, M5)	1 (M8)	3 (M7, M9, M10)
Bouncer	0	1 (M3)	6 (M1, M4, M5, M8, M9, M10)	3 (M2, M6, M7)	0	0
Messenger	4 (M2, M3, M4, M7)	6 (M1, M5, M6, M8, M9, M10)	0	0	0	0
Summarizer	4 (M3, M4, M6, M7)	6 (M1, M2, M5, M8, M9, M10)	0	0	0	0
Assistant	5 (M1, M3, M4, M7, M9)	4 (M2, M6, M8, M10)	1 (M5)	0	0	0
Gossip	0	0	1 (M8)	1 (M10)	7 (M1, M2, M4, M5, M6, M7, M9)	1 (M3)
Alien	0	5 (M3, M4, M5, M9, M10)	4 (M1, M2, M7, M8)	1 (M6)	0	0
Bureaucrat	1 (M3)	1 (M1)	6 (M2, M4, M5, M6, M8, M9)	1 (M7)	1 (M10)	0

**Table 5:** The extent to which the respondents experience the situated roles.

Frequency	Obstetricians	Midwives
Frequently	Summarizer; messenger; assistant	Summarizer; messenger; assistant; alien
Occasionally	Alien; wingman; third wheel; gossip	Accomplice; bureaucrat; bouncer
Rarely	Accomplice; bouncer; bureaucrat	Wingman; third wheel; gossip

#### Desired Frequency of the Situated Roles

To understand the desired frequency of each situated role, we analyzed the users’ responses for how frequently they would like to experience the roles. We looked at the midwives’ and obstetricians’ answers separately.

Tables 6 and 7 show the responses of the obstetricians and midwives respectively. The numbers in the table indicate the number of respondents that chose the option.

To better understand the extent to which different situated roles are wanted, we assigned them to 3 different categories:

1. Frequently: more than half of the respondents wanted the role to be experienced at least frequently.
2. Occasionally: more than half of the respondents wanted the role to be experienced at least occasionally.
3. Rarely: more than half of the respondents wanted to experience the role rarely at most.

The extent to which the respondents want to experience the situated roles is shown in Table 8.

**Table 6:** The desired frequency of experiencing the situated roles as reported by the obstetricians. Participant identifier listed in parentheses.

Situated role	Very frequently (n)	Frequently (n)	Occasionally (n)	Rarely (n)	Very rarely (n)	Not at all (n)
Wingman	0	3 (O2, O3, O5)	2 (O1, O4)	0	0	0
Accomplice	0	0	2 (O1, O5)	0	0	3 (O2, O3, O4)
Third wheel	0	0	0	1 (O2)	0	4 (O1, O3, O4, O5)
Bouncer	0	0	0	0	0	5 (O1, O2, O3, O4, O5)
Messenger	2 (O2, O3)	3 (O1, O4, O5)	0	0	0	0
Summarizer	2 (O2, O3)	3 (O1, O4, O5)	0	0	0	0
Assistant	0	5 (O1, O2, O3, O4, O5)	0	0	0	0
Gossip	0	0	3 (O1, O4, O5)	1 (O2)	1 (O3)	0
Alien	0	0	2 (O4, O5)	2 (O2, O3)	1 (O1)	0
Bureaucrat	0	0	1 (O5)	1 (O2)	1 (O1)	2 (O3, O4)

**Table 7:** The desired frequency of experiencing the situated roles as reported by the midwives. Participant identifier listed in parentheses.

Situated role	Very frequently (n)	Frequently (n)	Occasionally (n)	Rarely (n)	Very rarely (n)	Not at all (n)
Wingman	0	2 (M2, M5)	5 (M1, M3, M4, M6, M7)	1 (M8)	0	2 (M9, M10)
Accomplice	0	1 (M8)	4 (M1, M3, M6, M7)	2 (M4, M5)	2 (M2, M9)	1 (M10)
Third wheel	0	0	0	0	1 (M5)	9 (M1, M2, M3, M4, M6, M7, M8, M9, M10)
Bouncer	0	1 (M1)	1 (M8)	1 (M7)	1 (M2)	6 (M3, M4, M5, M6, M9, M10)
Messenger	5 (M2, M3, M4, M5, M7)	5 (M1, M6, M8, M9, M10)	0	0	0	0
Summarizer	8 (M2, M3, M4, M5, M6, M7, M8, M10)	2 (M1, M9)	0	0	0	0
Assistant	5 (M1, M2, M3, M4, M7)	4 (M6, M8, M9, M10)	1 (M5)	0	0	0
Gossip	0	0	0	0	6 (M1, M6, M7, M8, M9, M10)	4 (M2, M3, M4, M5)
Alien	0	0	2 (M4, M7)	4 (M1, M2, M6, M8)	4 (M3, M5, M9, M10)	0
Bureaucrat	1 (M8)	0	1 (M6)	3 (M1, M4, M7)	2 (M2, M9)	3 (M3, M5, M10)

**Table 8:** The extent to which the respondents want to experience the situated roles.

Frequency	Obstetricians	Midwives
Frequently	Summarizer; messenger; assistant; wingman	Summarizer; messenger; assistant
Occasionally	Gossip	Accomplice; wingman
Rarely	Alien; third wheel; bureaucrat; bouncer; accomplice	Alien; third wheel; bureaucrat; bouncer; gossip

We assumed that favorable roles are ones that users want to experience more frequently than they currently do. Unfavorable roles are ones that users want to experience less frequently than they currently do. Accordingly, the summarizer, the messenger, the assistant, and the wingman were regarded as favorable by both obstetricians and midwives. However, the obstetricians wanted to experience the wingman more frequently than the midwives. The alien, the third wheel, the bureaucrat, and the bouncer were regarded as unfavorable by both groups.

As for the gossip and accomplice, we considered them to be ambivalent. Their ideal frequency is the same as their current frequency. The midwives considered the gossip unfavorable, but the obstetricians wanted to experience it occasionally. Conversely, the obstetricians considered the accomplice unfavorable, but the midwives wanted to experience it occasionally.

#### Importance of the Situated Roles

As mentioned in the Methods section, for favorable roles, we asked the staff about the importance of their presence. For unfavorable roles, we asked about the importance of their absence. The accomplice role was treated as favorable to the prenatal care staff as it is created by them. The gossip role was treated as unfavorable as it denotes a lack of trust in the system.

To understand the importance of the situated roles, we looked at how much the respondents agreed that the roles’ presence or absence was important. We also looked at the midwives’ and obstetricians’ answers separately. Tables 9 and 10 show the responses of the obstetricians and midwives, respectively. The numbers in the table indicate the number of respondents that chose the option.

To better understand the importance of the different situated roles, we assigned them to 4 different categories:

1. Very important: at least half of the respondents strongly agree that the presence or absence of the role is important.
2. Important: at least half of the respondents agree that the presence or absence of the role is important.
3. Somewhat important: at least half of the respondents somewhat agree that the presence or absence of the role is important.
4. Not important at all: at least half of the respondents disagree that the presence or absence of the role is important.

The situated roles’ levels of importance are shown in Table 11.

**Table 9:** The extent to which the obstetricians agree that the situated roles are important. Participant identifier listed in parentheses.

Situated role	Strongly agree (n)	Agree (n)	Somewhat agree (n)	Disagree (n)
Presence of wingman	0	4 (O2, O3, O4, O5)	1 (O1)	0
Absence of accomplice	1 (O1)	1 (O5)	2 (O3, O4)	1 (O2)
Absence of third wheel	1 (O3)	4 (O1, O2, O4, O5)	0	0
Absence of bouncer	0	1 (O5)	1 (O3)	3 (O1, O2, O4)
Presence of messenger	4 (O1, O2, O3, O4)	1 (O5)	0	0
Presence of summarizer	4 (O1, O2, O3, O4)	1 (O5)	0	0
Presence of assistant	2 (O1, O2)	3 (O3, O4, O5)	0	0
Absence of gossip	2 (O2, O3)	3 (O1, O4, O5)	0	0
Absence of alien	5 (O1, O2, O3, O4, O5)	0	0	0
Absence of bureaucrat	1 (O4)	4 (O1, O2, O3, O5)	0	0

**Table 10:** The extent to which the midwives agree that the situated roles are important. Participant identifier listed in parentheses.

Situated role	Strongly agree (n)	Agree (n)	Somewhat agree (n)	Disagree (n)
Presence of wingman	1 (M5)	7 (M1, M2, M3, M4, M6, M7, M8)	2 (M9, M10)	0
Absence of accomplice	0	4 (M3, M5, M6, M8)	6 (M1, M2, M4, M7, M9, M10)	0
Absence of third wheel	8 (M1, M2, M3, M4, M5, M6, M8, M10)	2 (M7, M9)	0	0
Absence of bouncer	0	5 (M1, M2, M3, M5, M6)	3 (M4, M7, M8)	2 (M9, M10)
Presence of messenger	10 (M1, M2, M3, M4, M5, M6, M7, M8, M9, M10)	0	0	0
Presence of summarizer	10 (M1, M2, M3, M4, M5, M6, M7, M8, M9, M10)	0	0	0
Presence of assistant	5 (M1, M2, M3, M4, M7)	5 (M5, M6, M8, M9, M10)	0	0
Absence of gossip	6 (M1, M2, M3, M4, M5, M9)	4 (M6, M7, M8, M10)	0	0
Absence of alien	10 (M1, M2, M3, M4, M5, M6, M7, M8, M9, M10)	0	0	0
Absence of bureaucrat	6 (M1, M3, M5, M6, M7, M8)	4 (M2, M4, M9, M10)	0	0

**Table 11:** The situated roles’ levels of importance.

Importance	Obstetricians	Midwives
Very important	Presence of summarizer; presence of messenger; presence of assistant; absence of alien	Presence of summarizer; presence of messenger; presence of assistant; absence of alien; absence of third wheel; absence of bureaucrat; absence of gossip
Important	Presence of wingman; absence of third wheel; absence of bureaucrat; absence of gossip	Presence of wingman
Somewhat important	Presence of accomplice	Presence of accomplice; absence of bouncer
Not important at all	Absence of bouncer	—^a^

^a^Not applicable.

## Discussion

### Principal Findings

We identified 10 situated roles that EMR systems play in Japanese prenatal care. On the basis of the feedback of the obstetricians and the midwives, we validated the roles and understood the users’ wants and priorities regarding them.

**Figure 4:**
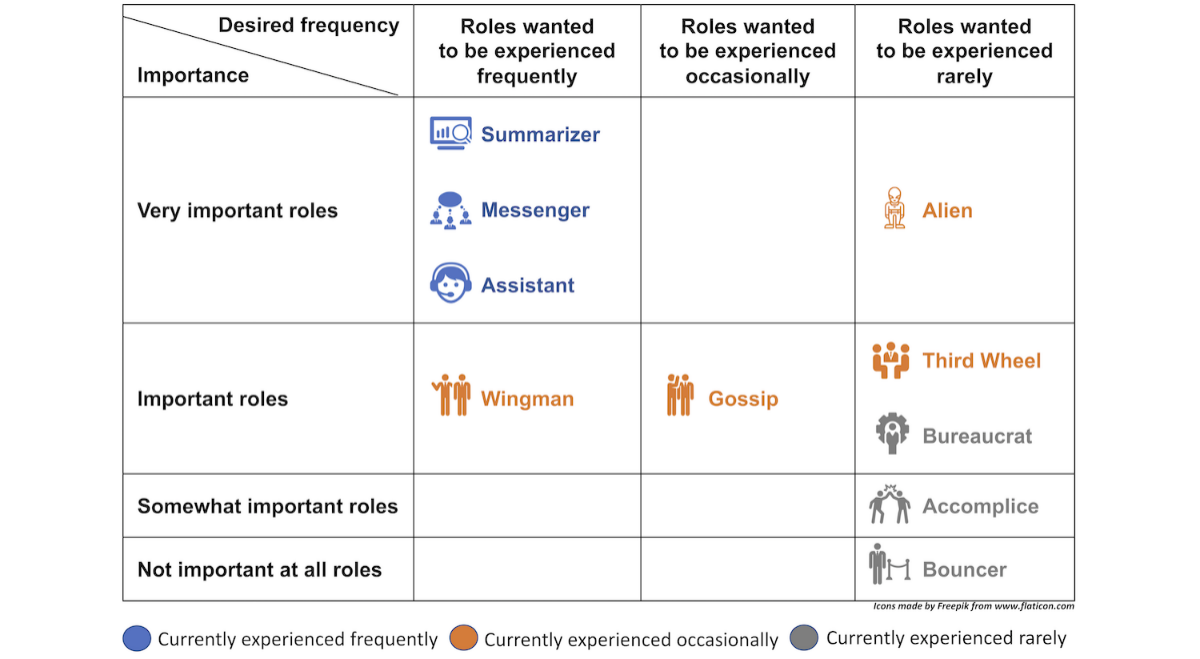
The situated roles of the electronic medical record system for the obstetricians.

**Figure 5:**
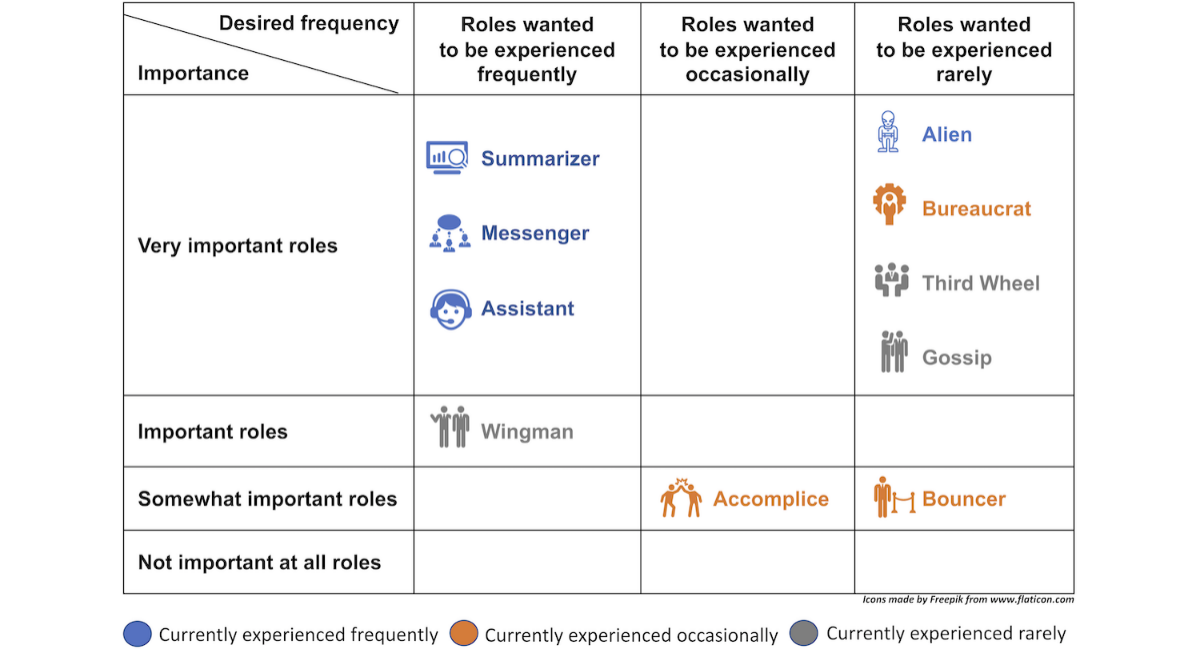
The situated roles of the electronic medical record system for the midwives.

Our results offer multiple EMR redesign opportunities in prenatal care. Figures 4 and 5 can serve as redesign guidelines for satisfying the wants of the obstetricians and the midwives, respectively.

To improve the usability and usefulness of the EMR systems, designers can amplify the favorable roles (the roles wanted to be experienced frequently) and minimize the unfavorable roles (the roles wanted to be experienced rarely). To align their design activities with the priorities of the users, designers can focus on the roles reported as important by the users. To increase the impact of their redesigns, the designers can focus on minimizing unfavorable roles that are frequently experienced, for example, the alien, or amplifying favorable roles that are less frequently experienced, for example, the wingman.

The gossip and the accomplice roles seem to be ambivalent. The midwives want to clearly document sensitive psychosocial information in most cases, whereas the obstetricians seem to be more hesitant. Conversely, the midwives want to use the EMR system occasionally to have a moment to think, whereas obstetricians prefer to rarely do so.

Through the gossip role, our study highlighted the challenge of documenting sensitive psychosocial information in EMR systems. A reason for not documenting such information could be that psychosocial information is viewed as *subjective* and sometimes *not legitimate enough* to be placed in a permanent medical record [38]. Conversely, when such information is documented, it may be documented in vague and ambiguous ways. This behavior may be attributed to the health care providers’ distrust in the security of EMR systems or their unfamiliarity with the laws governing the access and use of health care data. It is important to note that in Japan, one in every 20 women may experience domestic violence (DV) during pregnancy [39]. Furthermore, pregnant Japanese women want their psychosocial information to be documented in detail in their EMRs [40]. Therefore, clearly documenting sensitive information is needed to (1) respect the preferences of the pregnant women and (2) address psychosocial issues, such as DV, that are not easily disclosed or discussed [41]. Further investigations should be conducted to understand if the gossip role is a result of the EMR system’s design as it might create a feeling of distrust for the medical staff, or if it is a result of the medical staff’s uncertainty regarding the laws governing health care data.

As for the accomplice role, it is created by the staff to pause the conversation with the pregnant women. In this case, the reasons behind this role should be further understood as it presents a possible conflict between the needs of health care providers and patients and raises an important question: whose needs should we consider and prioritize when designing EMR systems?

Our results also suggest that the EMR system is viewed first and foremost as a tool for supporting the clinical workflow. The messenger, summarizer, assistant, and alien were very important situated roles for both user groups. These results shed light on *clear* redesign targets, ones that improve the EMR system’s capabilities as a tool to support the clinical process.

However, the EMR system’s roles as a tool that supports the communication with the pregnant women seems secondary, as shown through the bouncer role. Both midwives and obstetricians do not think it is important that the pregnant women see the EMR screen. Conversely, they think it is important to use the EMR screen as an explanation support tool. Further research is needed to understand the attitudes of the prenatal care providers regarding the EMR system as a communication support tool.

A previous study found no difference between the perceptions of physicians and nurses regarding EMR systems [13]. Even though the experiences and wants of the midwives and the obstetricians overlapped for certain situated roles (the messenger, the summarizer, the assistant, and the alien), they differed for others. Our results imply that the users’ experiences with the EMR system, and their aspirations regarding it, are related to the nature and purpose of their job. Aligning with previous findings in Japanese prenatal care settings [22], these results highlight the different responsibilities that obstetricians and midwives have in prenatal care, as it can be argued that the obstetricians’ focus is clinical while the midwives’ focus is more psychosocial. Moreover, in OB-led checkups, the midwife assists the obstetrician instead of being the main caretaker. Consequently, the pregnant woman’s chair faces the obstetrician’s desk. The desk placement in this case might affect the situated roles exhibited by the EMR system.

Accordingly, EMR redesign efforts should take into consideration not only the prenatal care context but also the different types of EMR users in this context. One direction to explore is having 2 different EMR systems—one for the midwives and another for the obstetricians—rather than having a general prenatal care EMR system. In this case, we need to ensure that the use of different systems does not hinder collaborative care [42].

### Limitations

The first limitation of this study is the number of observations. In addition, the obstetricians that we observed were all males. As the communication style may differ substantially depending on the obstetrician and pregnant woman, more situated roles may be identified through additional observations that include female obstetricians.

Moreover, our results may not be generalizable. The readers will have to assess the degrees to which our results can be transferred to health care or cultural settings of their interest.

In other respects, it would be valuable to quantify the number of occurrences relating to a situated role. Owing to the limited number of field observations that we were able to conduct, our analysis aimed to provide a purely qualitative and nuanced description of the situated roles, without counting the frequency of occurrences. Quantifying the occurrences is particularly valuable to evaluate redesign activities. To evaluate the success of a redesign activity, designers may need to objectively quantify and compare the frequency of certain occurrences in the pre-redesign and postredesign phases. To facilitate the redesign evaluation process, our future work will look into ways to automatically and objectively quantify the occurrences without the need for manual data analysis.

### Conclusions

By looking at how the prenatal care providers appropriated the EMR system, we identified 10 situated roles that EMR systems play in Japanese prenatal care settings. We also identified the wants and the priorities of the users regarding the EMR system’s situated roles.

We found that prenatal care providers mainly use the EMR system as a summarizer to have a quick summary of the pregnancy course, as a messenger to communicate patient information across staff rotations, and as an assistant to prepare for the checkups. They would like to use the EMR system as a tool to support their explanations to the pregnant women more frequently. We also found that the providers may use the EMR system as an accomplice to pause communication with the pregnant women. Interestingly, they do not think it is important for the pregnant women to view the EMR screen during the checkups. Our results further highlighted the lack of trust in the security of EMR systems and how it can negatively affect prenatal care provision.

In other respects, we found a difference in the experiences and attitudes of the obstetricians and the midwives regarding the use of the EMR system. These findings imply the need for different EMR system designs to better fit the job descriptions and tasks of the different user groups.

Our study serves as an example to show how EMR systems can be redesigned from appropriation. Our results could be used to redesign EMR systems to better fit the contextual needs of their users in prenatal care.

## Abbreviations

DV: domestic violence
EHR: electronic health record
EMR: electronic medical record
MCH: maternal and child health
MW-led: midwife-led
OB-led: obstetrician-led
